# Calculation of Robot Multi-Fingered Grasping Force and Displacement Based on the Newton–Subgradient Non-Smooth Greedy Randomized Kaczmarz Method for Solving Linear Complementarity Problem

**DOI:** 10.3390/s25072309

**Published:** 2025-04-05

**Authors:** Zhiwei Ai, Chenliang Li

**Affiliations:** 1 School of Mathematics and Computing Science, Guilin University of Electronic Technology, Guilin 541004, China; aizhiwei752@163.com; 2 School of Artificial Intelligence, Guilin University of Aerospace Technology, Guilin 541004, China; 3 Center for Applied Mathematics of Guangxi (GUET), Guilin 541004, China; 4 Guangxi Colleges and Universities Key Laboratory of Data Analysis and Computation, Guilin 541004, China

**Keywords:** multi-fingered grasping problem, linear complementarity problem, subgradient, non-smooth system of equations, Kaczmarz method

## Abstract

The calculation of grasping force and displacement is important for multi-fingered stable grasping and research on slipping damage. By linearizing the friction cone, the robot multi-fingered grasping problem can be represented as a linear complementarity problem (LCP) with a saddle-point coefficient matrix. Because the solution methods for LCP proposed in the field of numerical computation cannot be applied to this problem and the Pivot method can only be used for solving specific grasping problems, the LCP is converted into a non-smooth system of equations for solving it. By combining the Newton method with the subgradient and Kaczmarz methods, a Newton–subgradient non-smooth greedy randomized Kaczmarz (NSNGRK) method is proposed to solve this non-smooth system of equations. The convergence of the proposed method is established. Our numerical experiments indicate its feasibility and effectiveness in solving the grasping force and displacement problems of multi-fingered grasping.

## 1. Introduction

Given that simple end-effectors cannot meet the demands of complex manipulation, multi-fingered dexterous hand grasping has become one of the most important topics in humanoid robot research. Research on the multi-fingered dexterous hand covers multiple aspects, including structural design [1,2,3], grasping mechanisms [4], grasping control [5,6], and intelligent operation through multi-sensor information fusion [7,8]. The core of these research topics is to ensure that the multi-fingered dexterous hand applies an appropriate grasping force to objects for stable grasping while avoiding damage [9,10]. There are three main reasons for damage during the grasping process: instability in the dexterous hand’s grasp, causing the object to fall; excessive grasping force from the dexterous hand, resulting in crushing damage to the object; slippage displacement between the dexterous hand and the object causing surface damage. The first two damage mechanisms primarily relate to grasping force, whereas the third involves both grasping force and displacement. Therefore, accurate calculation of both the grasping force and the corresponding displacement is critical.

Because the relationship between the multi-fingered dexterous hand and the object presents variable contact conditions during the grasping process [11], it poses a challenge to the calculation of grasping forces and displacements in the robot multi-fingered grasping problem. To solve this challenge, researchers have introduced complementarity relationships. The complementarity methods for the two-dimensional (2D) multi-fingered grasping problem are relatively mature. Various complementarity methods, such as the Pivot method [12,13], can be used to solve it efficiently. However, the complementarity method for the three-dimensional (3D) grasping problem is far from mature. Because the 3D multi-fingered grasping problem can be modeled into different models based on different treatments of the friction contact relationship, complementarity methods of these models vary significantly.

Due to the nonlinearity of frictional contact, some researchers have modeled the multi-fingered grasping problem as an optimal problem with nonlinear complementarity constraints for its solution. For instance, the Bullet simulator [14] proposes a Projected Gauss–Seidel method to solve this optimization problem. Carpentier [15] combines the Alternating Direction Method of Multipliers with the Proximal Method to solve it. However, these methods all require solving subproblems in each outer iteration.

By using a polyhedral pyramid to approximate the Coulomb friction cone, some researchers have modeled the multi-fingered grasping problem as an LCP. For instance, Fahed modeled frictionless multi-fingered grasping [12], friction-considered hard finger grasping [16], and soft hand grasping [17] as LCPs. Panagiotopoulos [18] decomposed the multi-fingered grasping problem into a contact problem with known contact force, then modeled it as an LCP. Maximilian [19] modeled the multi-fingered grasping problem as an LCP and then transformed the LCP into an optimization problem for numerical solution. Wu and Li [20] transformed the elastic contact mechanics problem into an LCP with the Toeplitz coefficient matrix.

Numerous methods can be used to solve LCPs in numerical computation fields. For example, iterative methods, such as the projected method [21], the modular iteration method [22], and the Newton method [23,24], can be used to solve large-scale problems. The projected method and the modular iteration method have rapidly developed since Bai [25] introduced the idea of matrix splitting. But the convergence of these iterative methods requires that the coefficient matrix has a special structure, such as H-matrices [26], H^+^-matrices [27], and L-matrices [28]. However, the coefficient matrix of LCP arising from the 3D multi-fingered grasping problem is a saddle-point matrix, and existing numerical methods are unable to solve the problem; meanwhile, the Pivot method is only suitable for special cases.

Addressing the aforementioned issues, we utilize a merit function such that solving the LCP is equivalent to solving the non-smooth system of equations. In the process of seeking methods to solve non-smooth system of equations, it was found that the Kaczmarz method has advantages. On the one hand, Kaczmarz-type methods have demonstrated superior performance in solving linear [29,30] and nonlinear [31,32] systems of equations. On the other hand, the Kaczmarz method can just find the solution closest to the initial iteration value. These advantages motivated us to apply the Kaczmarz method to the solution of grasping problems. Based on this, we propose the NSNGRK method. If the NSNGRK method converges and the zero vector is set as the initial value, the result must be the solution to the multi-fingered grasping problem, even though there are multiple satisfactory grasping forces. Notably, in practical applications, state-of-the-art multi-fingered robotic systems can be equipped with embedded 6-DOF F/T sensors at each fingertip for real-time contact force monitoring. This sensory feedback can serve as critical input to the NSNGRK method for the online computation of external wrenches.

A brief overview of this paper is presented below. Firstly, the LCP model of the robot multi-fingered grasping problem is formulated based on the existing model. Secondly, the NSNGRK method is designed. Thirdly, the convergence of NSNGRK is proven. Fourthly, numerical examples are used to verify the feasibility and effectiveness of the NSNGRK method in solving multi-fingered grasping problems. The conclusions are briefly presented in the final section.

## 2. LCP Model of Multi-Fingered Grasping

**Figure 1:**
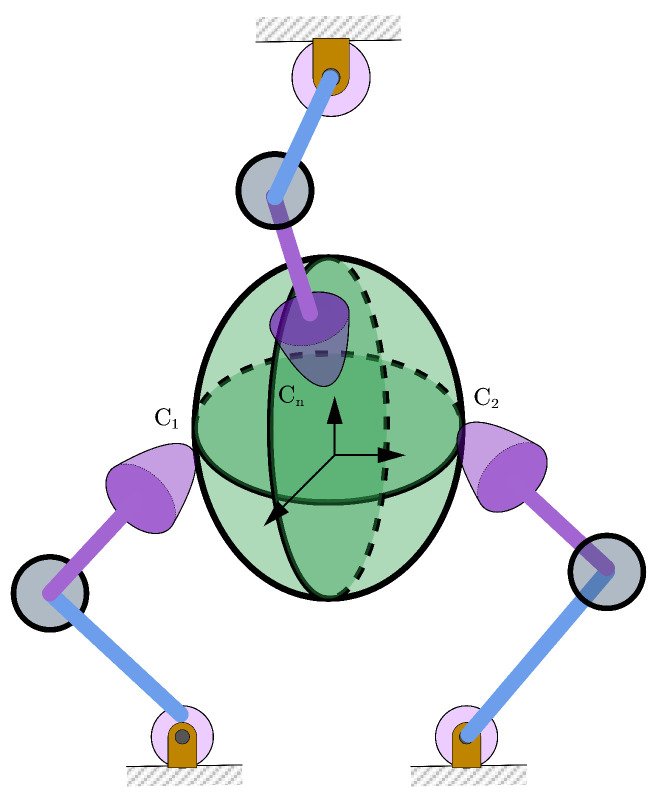
A multi-fingered hand grasping an object with the fingertips only.

**Figure 2:**
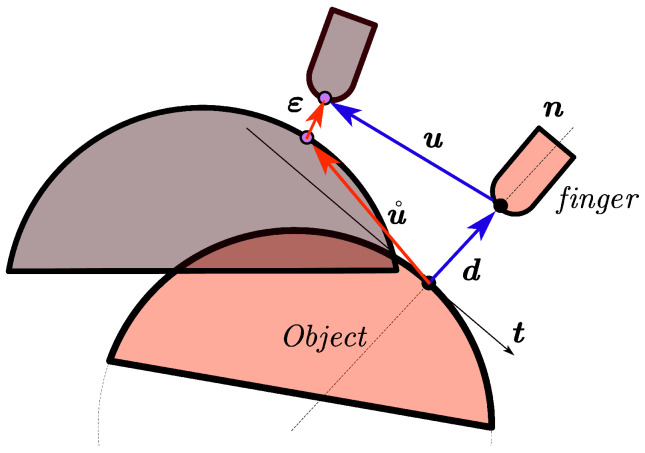
Schematic diagram of normal compatibility conditions.

Figure 1 illustrates a simple scenario in which a multi-fingered hand, equipped with n two-jointed fingers, grasps an object within a 3D space. Many more complex grasping scenarios can also be simplified into the form illustrated in this figure for further analytical studies. Consider a grasping case where finger *i* of the multi-fingered hand can exert a fingertip force on the object within the friction cone at the contact point, Fahed [16] and Panagiotopoulos [18] transformed the grasping problem into a linear complementarity problem. For the grasped object, when a finger applies a normal force to the object, a friction force arises between the finger and object under external loading. This friction force combines with the normal force to balance external forces. On the finger, a tangential reaction force develops due to friction. When the grasp achieves force closure, there is

(1) $$G_{N}r_{N}+G_{T}r_{T}=F_{ext},$$

where $r_{N}$ denotes the normal force, $r_{T}$ the tangential force, $G_{N}$ the grasping matrix for the normal force, $G_{T}$ the grasping matrix for the tangential force, and $F_{ext}$ the external wrench. According to the principle of virtual work [33], we have

(2) $$\begin{array}{cc} {\overset{˚}{u}}_{N} & =-G_{N}^{T}\overset{˚}{u},\\ {\overset{˚}{u}}_{T} & =-G_{T}^{T}\overset{˚}{u}, \end{array}$$

where $\overset{˚}{u}$, ${\overset{˚}{u}}_{N}$, and ${\overset{˚}{u}}_{T}$ denote the displacement, the normal displacement, and the tangential displacement of the object. According to the unilateral contact condition, in the normal direction, there exists

(3) $$\varepsilon_{N}=u_{N}-{\overset{˚}{u}}_{N}+d_{N},$$

where $\varepsilon_{N}$ denotes the normal slippage displacement, $u_{N}$ the normal displacement of the fingertip, and $d_{N}$ the initial normal distance vector between the fingers and the object. The compatibility conditions of unilateral contact behavior can be described as in Figure 2. The complementarity relationship between the normal slippage displacement $\varepsilon_{N}$ and the normal force $r_{N}$ can be written as follows:

(4) $$\varepsilon_{N}^{T}r_{N}=0,\varepsilon_{N}\geq 0,r_{N}\geq 0.$$

Similarly, the tangential boundary condition can be obtained as follows:

(5) $$\varepsilon_{T}=u_{T}-{\overset{˚}{u}}_{T},$$

where $\varepsilon_{T}$ denotes the tangential slippage displacement, and $u_{T}$ denotes the tangential displacement of the fingertip. According to Coulomb’s friction law, when contact occurs, there exists

(6) $$\begin{array}{cc} \gamma_{i} & =\mu \left|r_{Ni}\right|-\left|r_{Ti}\right|,i=1,2,\ldots ,n,\\ \gamma_{i} & \geq 0, \end{array}$$

where μ denotes the frictional coefficient, and *n* denotes the number of times contact is made. The frictional condition can be described as follows:

If the tangential force is less than the friction force (i.e., there exists γ>0), the tangential slippage displacement $y_{Ti}$ equals zero.

If the tangential force is equal to the friction force (i.e., there exists γ=0), the tangential slippage displacement $y_{Ti}$ is non-zero.

The direction of the slippage displacement is opposite to that of the tangential force. Equation (6) represents the Coulomb friction cones, each of which is a three-dimensional cone. To linearize the friction cone, a polyhedral cone is used to approximate the friction cone. The equation for the friction cone is

(7) $$C_{e}=\left\{\left(r_{n},r_{o},r_{t}\right)|f=\sqrt{\left({r_{o}}^{2}+{r_{t}}^{2}\right)}-\mu r_{n}\leq 0,r_{n}\geq 0\right\},$$

when the friction cone is approximated by a pyramid with *m* faces, the edges of the pyramid can be expressed as ${\left[\begin{array}{ccc} r_{n}, & \mu_{i}r_{n}\cos\alpha_{i}, & \mu_{i}r_{n}\sin\alpha_{i} \end{array}\right]}^{T},i=1,\ldots ,m$ in the contact coordinate system $\left\{n,o,t\right\}$. The piecewise-linearized form of Equation (7) becomes

(8) $$\begin{array}{c} C_{p}=\left\{\left(r_{n},r_{o},r_{t}\right)|-r_{o}\cos\alpha_{i}-r_{t}\sin\alpha_{i}+\mu r_{n}\geq 0,r_{n}\geq 0\right\}, \end{array}$$

where $\mu_{i}$ is the frictional coefficient. By substituting (8) into (6), we obtain

(9) $$\begin{array}{c} \gamma_{i}=\left[\begin{array}{ccc} \mu_{i} & -\cos\alpha_{1} & -\sin\alpha_{1}\\ \vdots & \vdots & \vdots \\ \mu_{i} & -\cos\alpha_{m} & -\cos\alpha_{m} \end{array}\right]\left[\begin{array}{c} r_{n}\\ r_{o}\\ r_{t} \end{array}\right]. \end{array}$$

If $T_{Ni}=\left[\begin{array}{c} \mu_{i}\\ \vdots \\ \mu_{i} \end{array}\right],T_{Ti}=\left[\begin{array}{c} -\cos\alpha_{1}\\ \vdots \\ -\cos\alpha_{m} \end{array}\begin{array}{c} -\sin\alpha_{1}\\ \vdots \\ -\sin\alpha_{m} \end{array}\right]$, then Equation (9) can be rewritten as

(10) $$\gamma =T_{N}r_{N}+T_{T}r_{T}\;\;,$$

where

$$\begin{array}{cc} T_{N} & =blkdiag\left[T_{N1},\ldots ,T_{Nn}\right],\\ T_{T} & =blkdiag\left[T_{T1},\ldots ,T_{Tn}\right], \end{array}$$

in which blkdiag denotes a block diagonal. Given that the normal force is orthogonal to the tangential motion, let λ be a coefficient related to the tangential motion satisfying the following relationship:

(11) $$\varepsilon_{T}=T_{T}\lambda ,\lambda \geq 0.$$

Then, γ and λ are orthogonal, that is,

(12) $$\gamma^{T}\lambda =0.$$

If linear elastic behavior exists between the finger and the object during the grasping, there is

(13) u=Er,

where

$$\begin{array}{c} u=\left[\begin{array}{c} u_{N}\\ u_{T} \end{array}\right],r=\left[\begin{array}{c} r_{N}\\ r_{T} \end{array}\right],E=\left[\begin{array}{cc} E_{N} & 0\\ 0 & E_{T} \end{array}\right], \end{array}$$

and E denotes the flexibility matrix.

Since the grasping force cannot be unbounded, to further constrain the solution set we impose an upper bound ${\bar{r}}_{N}$ on the normal force component, yielding

(14) $$\begin{array}{c} e^{T}r_{N}\leq {\bar{r}}_{N} \end{array},$$

where $e={\left[1,\ldots ,1\right]}^{T}$. By introducing a non-negative relaxation factor $\bar{w}$, Equation (14) can be rewritten as

(15) $$\begin{array}{c} -e^{T}r_{N}+{\bar{r}}_{N}=\bar{w} \end{array}.$$

At the same time, the normal unilateral contact conditions (3) can be rewritten as

(16) $$\begin{array}{c} \varepsilon_{N}=u_{N}-{\overset{˚}{u}}_{N}+d_{N}+e^{T}\bar{z} \end{array}$$

where $\bar{w}$ and $\bar{z}$ are complementarity variables. When the normal forces are lower than the upper bound (i.e., $e^{T}r_{N}<\bar{r_{N}}$), we have $\bar{w}>0,\bar{z}=0$; the unilateral contact conditions remain unchanged. When the normal forces reach the upper bound (i.e., $e^{T}r_{N}=\bar{r_{N}}$), we have $\bar{w}=0,\bar{z}>0$; the unilateral contact conditions will depend on $\bar{z}$. The unilateral contact condition will change in this situation, which makes disables the normal force from reaching the upper bound. Let

$$C=\left[\begin{array}{cc} E_{T} & G_{T}^{T}\\ G_{T} & 0 \end{array}\right].$$

If C is a nonsingular matrix, then

$$C^{-1}=\left[\begin{array}{cc} 0 & A^{T}\\ A & U \end{array}\right],$$

where

$$\begin{array}{c} A^{T}={E_{T}}^{-1}{G_{T}}^{T}{\left(G_{T}{E_{T}}^{-1}{G_{T}}^{T}\right)}^{-1},\\ U=-{\left(G_{T}{E_{T}}^{-1}{G_{T}}^{T}\right)}^{-1}. \end{array}$$

A standard LCP can be obtained from the relationship between (1), (2), (4), (5), (10)–(13), (15), and (16), which can be written as

(17) $$w-\mathrm{Mz}=q,w\geq 0,z\geq 0,w^{T}z=0,$$

where   

$w=\left[\begin{array}{c} \varepsilon_{N}\\ \gamma \\ \bar{w} \end{array}\right],$ $z=\left[\begin{array}{c} r_{N}\\ \lambda \\ \bar{z} \end{array}\right],$ $q=\left[\begin{array}{c} d_{N}+G_{N}^{T}\mathrm{UP}\\ T_{T}^{T}A^{T}F_{ext}\\ {\bar{r}}_{N} \end{array}\right]$, $M=\left[\begin{array}{ccc} E_{N}-G_{N}^{T}{\mathrm{UG}}_{N} & G_{N}^{T}{\mathrm{AT}}_{T} & e^{T}\\ T_{N}^{T}-T_{T}^{T}A^{T}G_{N} & 0 & 0\\ -e^{T} & 0 & 0 \end{array}\right]$.

It is apparent that the coefficient matrix *M* in Formula (17) is neither symmetric nor skew-symmetric and the element in the bottom right corner is a zero matrix. This type of matrix is also known as a saddle-point matrix. Since the coefficient matrix *M* is a saddle-point matrix, methods like modulus based splitting methods and projected methods cannot be applied, as a saddle-point matrix does not satisfy their convergence conditions. As mentioned before, the Pivot method, as detailed in Algorithm 1, provides an effective solution for linear complementarity problems arising from 2D grasping problems. When reducing 3D grasping problems to 2D equivalents, the resulting linear complementarity problem’s coefficient matrix becomes a skew-symmetric saddle-point matrix. As the Pivot method’s convergence criterion requires copositive-plus matrices [13], convergence is guaranteed in this configuration. However, for 3D grasping problems where the coefficient matrix, often fails to satisfy copositive conditions, the Pivot method remains applicable only in specific scenarios.
**Algorithm 1 **Pivot method [13]**Require:** 
Coefficient matrix $M\in R^{n\times n}$, vector $q\in R^{n}$**Ensure:** 
Solution vector pair (w,z)  1:**Check Initial Condition:** If q≥0, stop the calculation, (w,z)=(q,0) is a complementary basic feasible solution; otherwise, Otherwise, a column vector e of ones and an artificial variable $z_{0}$ must be introduced to construct a new system$$\left\{\begin{array}{c} w-\mathrm{Mz}-ez_{0}=q\\ w,z\geq 0,z_{0}\geq 0\\ w^{T}z=0 \end{array}\right.$$  2:**Formulate Tableau and Initialize Pivot:** Represent the system in a tabular format as $\left[I,-M,e,q\right]$. Determine$$-q_{s}=\max\left\{-q_{i}|i=1,\ldots ,m+n\right\},$$
take row *s* as the pivot row and the column corresponding to $z_{0}$ as the pivot column, perform pivot elimination, and set $y_{s}=z_{s}$.  3:**Determine Pivot Direction:** In the current tableau, let the column corresponding to variable $y_{s}$ be $d_{s}$. If $d_{s}\leq 0$, stop the calculation, obtaining an extreme direction of the feasible region of the problem; otherwise, determine the index *r* by the minimum ratio test such that$$\frac{{\bar{q}}_{r}}{d_{rs}}=\min\left\{\frac{{\bar{q}}_{i}}{d_{is}}|d_{is}>0\right\}.$$If the basic variable in row *r* is $z_{0}$, proceed to Step 4; otherwise, proceed to Step 3.  4:**Pivot Operation for Basic Variables:** Let the basic variable in row *r* be $w_{l}$ or $z_{l}$ (l≠s). Variable $y_{s}$ enters the basis, with row *r* as the pivot row and the column corresponding to $y_{s}$ as the pivot column, perform pivot elimination. If the leaving variable is $w_{l}$, set $y_{s}=z_{l}$; if the leaving variable is $z_{l}$, set $y_{s}=w_{l}$, then return to Step 2.  5:**Final Pivot to Remove Artificial Variable:** Variable $y_{s}$ enters the basis, and $z_{0}$ leaves the basis. Take row *r* as the pivot row and the column corresponding to $y_{s}$ as the pivot column, perform pivot elimination. Obtain a complementary basic feasible solution and stop the calculation.

## 3. Newton–Subgradient Non-Smooth Greedy Randomized Kaczmarz Method

In this section, we attempt to construct a new method to solve the LCP with a saddle-point coefficient matrix. Firstly, we transform the LCP into a non-smooth system of equations. There are two transformation ideas. One is to transform the LCP into absolute value equations (AVEs) like the modular matrix splitting iterative method. However, as shown in [34], determining the existence of a solution to AVEs is NP-hard. We choose the second transformation, that is, transforming the LCP into an equivalent non-smooth system of equations by introducing the Fischer–Burmeister (F-B) function. The F-B function, being semi-smooth and continuously differentiable except at the origin, yields the following non-smooth system of equations:

(18) $$\Phi \left(z\right)=\left[\begin{array}{c} \phi_{1}\left(z_{1},w_{1}\right)\\ \vdots \\ \phi_{n}\left(z_{n},w_{n}\right) \end{array}\right]=0,$$

where the *n*-dimensional real vector $z={\left[z_{1},\ldots ,z_{n}\right]}^{T}$, $\phi_{i}\left(z_{i},w_{i}\right)=\sqrt{z_{i}^{2}+w_{i}^{2}}-z_{i}-w_{i},w_{i}={\left(\mathrm{Mz}+q\right)}_{i},i=1,2,\ldots ,n$. Since the F-B function $\phi_{i}$ is locally Lipschitz-continuous [35], the subgradient of $\phi_{i}$ at z meets

$$g_{i}\left(z\right)\in \partial \phi_{i}\left(z\right),$$

where $\partial \phi_{i}\left(z\right)$ denotes the subdifferential. Thus, Equation (18) has a generalized Jacobian matrix $J_{C}\left(z\right)$ in the sense of Clarke [36] at z, denoted as

(19) $$J_{C}\left(z\right)=\left[\begin{array}{c} g_{1}^{T}\left(z\right)\\ \vdots \\ g_{m}^{T}\left(z\right) \end{array}\right]≜\frac{\Phi \left(z+h\right)-\Phi \left(z\right)}{h},$$

where *h* is a small disturbance. We can deduce that the iterative format of the Kaczmarz method for the non-smooth system of Equation (18) is

(20) $$z^{\left(k+1\right)}=z^{\left(k\right)}-\frac{\phi_{i}\left(z^{\left(k\right)}\right)}{\|g_{i}\left(z^{\left(k\right)}\right){\|}_{2}^{2}}g_{i}\left(z^{\left(k\right)}\right).$$

To solve the non-smooth system of equations by using the idea of the greedy randomized Kaczmarz method, it is necessary to construct a non-empty indicator set $U_{k}$ that guarantees a higher probability of selecting a row with larger residuals at the *k*th iteration. With the generalized Jacobian matrix $J_{C}\left(z^{\left(k\right)}\right)$, the idea of solving linear equations in reference [32] can be used to construct a non-empty set $U_{k}$ by setting a threshold $\epsilon_{k}$ related to the current iteration in a non-smooth system of equations

(21) $$\begin{array}{cc} \epsilon_{k} & =\frac{1}{2}\frac{1}{\|\Phi \left(z^{\left(k\right)}\right){\|}_{2}^{2}}\max_{1\leq i_{k}\leq m}\left\{\frac{{\left|\phi_{i_{k}}\left(z^{\left(k\right)}\right)\right|}^{2}}{\|J_{C}\left(i_{k},:\right){\|}_{2}^{2}}\right\}\\ \; & +\frac{1}{2}\frac{1}{\|J_{C}\left(z^{\left(k\right)}\right){\|}_{F}^{2}}. \end{array}$$

The indicator set is

(22) $$U_{k}=\left\{i_{k}|{\left|\phi_{i_{k}}\left(z^{\left(k\right)}\right)\right|}^{2}\geq \epsilon_{k}\|\Phi \left(z^{\left(k\right)}\right){\|}_{2}^{2}{\left\|g_{i_{k}}\left(z^{\left(k\right)}\right)\right\|}_{2}^{2}\right\}.$$

It is easy to prove that the indicator set $U_{k}$ is non-empty. Let us define the residual as follows:

(23) $${\tilde{r}}_{k}^{\left(i\right)}=\left\{\begin{array}{c} \phi_{i}\left(z^{\left(k\right)}\right),i\in U_{k}\\ 0,otherwise. \end{array}\right.$$

Then, the hyperplane is selected according to the probability $P\left(row=i_{k}\right)=\frac{{\left|{{\tilde{r}}_{k}}^{\left(i_{k}\right)}\right|}^{2}}{\|{\tilde{r}}_{k}{\|}_{2}^{2}},i_{k}\in U_{k}$. Now, we give the NSNGRK method for solving the LCP (please see Algorithm 2). Facchinei and Soares [37] demonstrated that although the F-B function is semi-smooth, its natural merit function is continuously differentiable. This allows the selection of any initial point during iteration. Specifically, when the initial iterate for the Kaczmarz method is set to the zero vector, the NSNGRK method, if convergent, will certainly converge to the point closest to the origin.
**Algorithm 2 **Newton–subgradient Non-smooth Greedy Randomized Kaczmarz (NSNGRK)**Require:** 
Coefficient matrix $M\in R^{n\times n}$, vector $q\in R^{n}$, precision threshold ϵ>0, initial value $z^{\left(0\right)}$**Ensure:** 
Solution vector $z^{*}$  1:Initialize residual $r=\Phi (z^{\left(0\right)})$  2:**for **i=1 **to** *n* **do**  3:      Compute local functions:$$w_{i}\leftarrow M\left(i,:\right)z^{\left(k\right)}+q_{i}$$$$\phi_{i}\left(z\right)\leftarrow \sqrt{z_{i}^{2}+w_{i}^{2}}-z_{i}-w_{i}$$  4:**end for**  5:**while **${∥r∥}_{2}\geq \epsilon$** do**  6:      Compute subgradient matrix:$$J_{C}\left(z^{\left(k\right)}\right)\leftarrow \frac{\Phi \left(z^{\left(k\right)}+h\right)-\Phi \left(z^{\left(k\right)}\right)}{h}\;\left(h=10^{-6}\right)$$  7:      Determine threshold $\epsilon_{k}$ (21) and active index set $U_{k}$ (22)  8:      Build probability distribution:$$P\left(i_{k}\right)\propto |r_{k}^{\left(i_{k}\right)}{|}^{2}/{∥r_{k}∥}_{2}^{2},\;\forall i_{k}\in U_{k}$$  9:      Update iteration:$$z^{\left(k+1\right)}\leftarrow z^{\left(k\right)}-\frac{\phi_{i_{k}}\left(z^{\left(k\right)}\right)}{∥g_{i_{k}}\left(z^{\left(k\right)}\right){∥}_{2}^{2}}g_{i_{k}}\left(z^{\left(k\right)}\right)$$  10:    Compute new residual $r\leftarrow -\Phi (z^{\left(k+1\right)})$  11:**end while**

## 4. Convergence Analysis

**Definition** **1** ([31])**.** *If for any $i\in \left\{1,2,\ldots ,m\right\}$ and $z_{1},z_{2}\in R^{n}$, there exists $\eta_{i}\in \left[0,\eta \right)$, $\eta =\max_{i}\eta_{i}<1/2$ such that*

$$\begin{array}{c} |\phi_{i}\left(z_{1}\right)-\phi_{i}\left(z_{2}\right)-\nabla \phi_{i}\left(z_{1}\right)\left(z_{1}-z_{2}\right)|\\ \leq \eta_{i}\left|\phi_{i}\left(z_{1}\right)-\phi_{i}\left(z_{2}\right)\right|, \end{array}$$

*then the function $\Phi :R^{n}\to R^{m}$ satisfies the local tangential cone condition.*

**Lemma** **1** ([36])**.** *The F-B function is a convex function, and it is smooth at any point except the origin.*

**Lemma** **2** ([32])**.** *If a matrix $A\in R^{m\times n}$ has a full column rank, we have ${\left\|\mathrm{Az}\right\|}_{2}^{2}\geq \lambda_{min}\left(A^{T}A\right){\left\|z\right\|}_{2}^{2},\forall z\in R^{n}$.*

**Lemma** **3.** *If the non-smooth convex function $\phi \left(z\right)$ satisfies the local tangential condition at point $z_{2}$, and the gradient of $\phi \left(z\right)$ at point $z_{2}$ is in the subdifferential of $\phi \left(z\right)$ at point $z_{1}$, then there exists a subgradient of $\phi \left(z\right)$ at point $z_{1}$$g\left(z_{1}\right),\eta \in \left[0,\eta \right)$, η=maxη<1/2, such that*

$$\begin{array}{c} |\phi_{i}\left(z_{1}\right)-\phi_{i}\left(z_{2}\right)-g_{i}\left(z_{1}\right)\left(z_{1}-z_{2}\right)|\\ \leq \eta_{i}\left|\phi_{i}\left(z_{1}\right)-\phi_{i}\left(z_{2}\right)\right|. \end{array}$$

**Proof.**  Because $\phi \left(z\right)$ satisfies the local tangential cone condition at point $z_{2}$, we have

$$\begin{array}{c} \left|\phi \left(z_{2}\right)-\phi \left(z_{1}\right)-\nabla \phi \left(z_{2}\right)\left(z_{2}-z_{1}\right)\right|\\ \leq \eta \left|\phi \left(z_{2}\right)-\phi \left(z_{1}\right)\right|. \end{array}$$

If $h=z_{2}-z_{1}$, obviously, when $\nabla \phi \left(z_{2}\right)\in int\left(\partial \phi \left(z_{1}\right)\right)$, $g\left(z_{1}\right)h\geq \nabla \phi \left(z_{2}\right)h$; when $\nabla \phi \left(z_{2}\right)\notin int\left(\partial \phi \left(z_{1}\right)\right)$, $g\left(z_{1}\right)h\geq \nabla \phi \left(z_{2}\right)h$ holds true if $g\left(z_{1}\right)=\nabla \phi \left(z_{2}\right)$. That is to say, we can always choose $\nabla \phi (z_{2})$ as a subgradient of ϕ. Then, we can obtain

$$\begin{array}{cc} \phi \left(z_{2}\right) & -\phi \left(z_{1}\right)-g\left(z_{1}\right)\left(z_{2}-z_{1}\right)\\ \; & \leq \phi \left(z_{2}\right)-\phi \left(z_{1}\right)-\nabla \phi \left(z_{2}\right)\left(z_{2}-z_{1}\right). \end{array}$$

Notice that $\phi \left(z\right)$ is convex, so we obtain

$$\begin{array}{c} \phi \left(z_{2}\right)-\phi \left(z_{1}\right)-g\left(z_{1}\right)\left(z_{2}-z_{1}\right)\geq 0. \end{array}$$

Then, we have

(24) $$\begin{array}{cc} \; & \left|\phi \left(z_{1}\right)-\phi \left(z_{2}\right)-g\left(z_{1}\right)\left(z_{1}-z_{2}\right)\right|\\ \; & \leq \left|\phi \left(z_{2}\right)-\phi \left(z_{1}\right)-\nabla \phi \left(z_{2}\right)\left(z_{2}-z_{1}\right)\right|\;\;\\ \; & \leq \eta \left|\phi \left(z_{2}\right)-\phi \left(z_{1}\right)\right|. \end{array}$$

 □

With the conclusion of Lemma 3 and reference [31], the following lemma can be easily obtained and proven.

**Lemma** **4.** *If the non-smooth function $\Phi :R^{n}\to R^{m}$ satisfies the local tangential cone condition, then for i∈1,..,m and any $z_{1},z_{2}\in R^{n}$, we have*

(25) $$|\phi_{i}\left(z_{1}\right)-\phi_{i}\left(z_{2}\right)|\;\geq \frac{1}{1+\eta_{i}}\left|g_{i}\left(z_{1}\right)\left(z_{1}-z_{2}\right)\right|.$$

**Lemma** **5.** If the non-smooth function $\Phi :R^{n}\to R^{m}$ satisfies the local tangent cone condition, there exists $z^{*}$ such that $\Phi \left(z^{*}\right)=0$, and as follows from the iterative formula (20), we have

$$\begin{array}{cc} \|z^{k+1}-z^{*}{\|}_{2}^{2} & -\;\|z^{k}-z^{*}{\|}_{2}^{2}\\ \; & \leq -\left(1-2\eta_{i}\right)\frac{{\left|\phi_{i}\left(z^{k}\right)\right|}^{2}}{\|g_{i}\left(z^{k}\right){\|}_{2}^{2}}. \end{array}$$

**Theorem** **1.** Suppose the non-smooth function $\Phi :D\subset R^{n}\to R^{m}$ is defined in a bounded closed set D, and there exists $z^{*}$ such that $\Phi \left(z^{*}\right)=0$. If for any z∈D, the function $\Phi \left(z\right)$ has a generalized Jacobi matrix $J_{C}\left(z\right)$, then the NSNGRK method is convergent and

$$\begin{array}{cc} \; & E\|z^{k+1}-z^{*}{\|}_{2}^{2}\\ \; & \leq \left(1-\frac{\left(1-2\eta \right)\beta_{k}\lambda_{\min}\left(J_{C}^{T}J_{C}\right)}{2{\left(1+\eta \right)}^{2}}\right)E{\left\|z^{k}-z^{*}\right\|}_{2}^{2},\\ \; & k=0,1,\ldots \end{array}$$

**Proof.**  From Lemma 5, we have

$$\begin{array}{c} \|z^{k+1}-z^{*}{\|}_{2}^{2}\leq {\left\|z^{k}-z^{*}\right\|}_{2}^{2}-\left(1-2\eta \right)\frac{{\left|\phi_{i_{k}}\left(z^{k}\right)\right|}^{2}}{\|g_{i_{k}}\left(z^{k}\right){\|}_{2}^{2}}. \end{array}$$

By taking the mathematical expectation on both side of the above formula, we can obtain

(26) $$\begin{array}{cc} \; & E\|z^{k+1}-z^{*}{\|}_{2}^{2}\\ \; & \leq E\|z^{k}-z^{*}{\|}_{2}^{2}-\left(1-2\eta \right)E\frac{{\left|\phi_{i_{k}}\left(z^{k}\right)\right|}^{2}}{\|g_{i_{k}}\left(z^{k}\right){\|}_{2}^{2}}. \end{array}$$

Due to $i_{k}\in U_{k}$,

(27) $${\left|\phi_{i_{k}}\left(z^{k}\right)\right|}^{2}\geq \epsilon_{k}\|\Phi \left(z^{k}\right){\|}_{2}^{2}{\left\|g_{i_{k}}\left(z^{k}\right)\right\|}_{2}^{2}.$$

Placing (27) into (26), we can obtain

(28) $$\begin{array}{cc} \; & E\|z^{k+1}-z^{*}{\|}_{2}^{2}\\ \; & \leq E\|z^{k}-z^{*}{\|}_{2}^{2}-\left(1-2\eta \right)\epsilon_{k}\sum_{i_{k}}^{m}{\left|\phi_{i_{k}}\left(z^{k}\right)\right|}^{2}. \end{array}$$

Due to $\phi_{i_{k}}\left(z^{*}\right)=0$, on the one hand, from Lemma 4, we have

$$\begin{array}{cc} |\phi_{i_{k}}\left(z^{k}\right)| & =\;|\phi_{i_{k}}\left(z^{k}\right)-\phi_{i_{k}}\left(z^{*}\right)|\\ \; & \geq \frac{1}{1+\eta }\left|g_{i_{k}}\left(z^{k}\right)\left(z^{k}-z^{*}\right)\right|, \end{array}$$

and then, we can obtain

(29) $$\begin{array}{c} \sum_{i_{k}=1}^{m}|\phi_{i_{k}}\left(z^{k}\right){|}^{2}\geq \frac{1}{{\left(1+\eta \right)}^{2}}\left|\right|J_{C}\left(z^{k}\right)\left(z^{k}-z^{*}\right){\left|\right|}_{2}^{2}. \end{array}$$

On the other hand, suppose $\alpha_{k}=\sum_{i_{k}=1,i_{k}\neq i_{k}-1}^{m}\left|\right|g_{i_{k}}{\left|\right|}_{2}^{2}$; from the threshold condition (21), we can obtain

$$\begin{array}{c} \varepsilon_{k}\left|\right|J_{C}\left(z^{k}\right){\left|\right|}_{F}^{2}=\frac{1}{2}\frac{\max_{1\leq i_{k}\leq m}\left\{\frac{|\phi_{i_{k}}{|}^{2}}{\left|\right|g_{i_{k}}{\left|\right|}_{2}^{2}}\right\}}{\frac{\left|\right|\Phi^{k}{\left|\right|}_{2}^{2}}{\left|\right|J_{C}\left(z^{k}\right){\left|\right|}_{F}^{2}}}+\frac{1}{2}\\ =\frac{1}{2}\frac{\max_{1\leq i_{k}\leq m}\left\{\frac{|\phi_{i_{k}}{|}^{2}}{\left|\right|g_{i_{k}}{\left|\right|}_{2}^{2}}\right\}}{\frac{\sum_{i_{k}=1}^{m}|\phi_{i_{k}}{|}^{2}}{\left|\right|J_{C}\left(z^{k}\right){\left|\right|}_{F}^{2}}}+\frac{1}{2}\\ =\frac{1}{2}\frac{\max_{1\leq i_{k}\leq m}\left\{\frac{|\phi_{i_{k}}{|}^{2}}{\left|\right|g_{i_{k}}{\left|\right|}_{2}^{2}}\right\}}{\sum_{i_{k}=1}^{m}\frac{\left|\right|g_{i_{k}}{\left|\right|}_{2}^{2}}{\left|\right|J_{c}^{k}{\left|\right|}_{F}^{2}}\cdot \frac{|\phi_{i_{k}}{|}^{2}}{\left|\right|g_{i_{k}}{\left|\right|}_{2}^{2}}}+\frac{1}{2}\\ \geq \frac{1}{2}\frac{\max_{1\leq i_{k}\leq m}\left\{\frac{|\phi_{i_{k}}{|}^{2}}{\left|\right|g_{i_{k}}{\left|\right|}_{2}^{2}}\right\}}{\sum_{i_{k}=1,i_{k}\neq i_{k}-1}^{m}\frac{\left|\right|g_{i_{k}}{\left|\right|}_{2}^{2}}{\left|\right|J_{C}\left(z^{k}\right){\left|\right|}_{F}^{2}}\cdot \frac{|\phi_{i_{k}}{|}^{2}}{\left|\right|g_{i_{k}}{\left|\right|}_{2}^{2}}}+\frac{1}{2}\\ \geq \frac{1}{2}\frac{\left|\right|J_{C}\left(z^{k}\right){\left|\right|}_{F}^{2}}{\sum_{i_{k}=1,i_{k}\neq i_{k}-1}^{m}\left|\right|g_{i_{k}}{\left|\right|}_{2}^{2}}+\frac{1}{2}\\ =\frac{1}{2}\left(\frac{\left|\right|J_{C}\left(z^{k}\right){\left|\right|}_{F}^{2}}{\alpha_{k}}+1\right). \end{array}$$

Suppose $\beta_{k}=\frac{1}{\alpha_{k}}+\frac{1}{\left|\right|J_{C}\left(z^{k}\right){\left|\right|}_{F}^{2}}$; then, we can obtain

(30) $$\begin{array}{c} \varepsilon_{k}\geq \frac{1}{2}\beta_{k}. \end{array}$$

From (28) and (29), we have

(31) $$\begin{array}{c} \begin{array}{cc} \; & E\|z^{k+1}-z^{*}{\|}_{2}^{2}\leq E{\left\|z^{k}-z^{*}\right\|}_{2}^{2}\\ \; & -\frac{\left(1-2\eta \right)}{{\left(1+\eta \right)}^{2}}\epsilon_{k}{\left\|J_{C}\left(z^{k}\right)\left(z^{k}-z^{*}\right)\right\|}_{2}^{2}. \end{array} \end{array}$$

Due to $E\|z^{k}-z^{*}{\|}_{2}^{2}={\left\|z^{k}-z^{*}\right\|}_{2}^{2}$, from Lemma 2, (30), and (31), we have

$$\begin{array}{cc} \; & E\|z^{k+1}-z^{*}{\|}_{2}^{2}\\ \; & \leq E\|z^{k}-z^{*}{\|}_{2}^{2}-\frac{1-2\eta }{{\left(1+\eta \right)}^{2}}\epsilon_{k}{\left\|J_{C}\left(z^{k}\right)\left(z^{k}-z^{*}\right)\right\|}_{2}^{2}\\ \; & \leq E\|z^{k}-z^{*}{\|}_{2}^{2}-\frac{\left(1-2\eta \right)\beta_{k}\lambda_{\min}\left(J_{C}^{T}J_{C}\right)}{2{\left(1+\eta \right)}^{2}}{\left\|z^{k}-z^{*}\right\|}_{2}^{2}\\ \; & \leq \left(1-\frac{\left(1-2\eta \right)\beta_{k}\lambda_{\min}\left(J_{C}^{T}J_{C}\right)}{2{\left(1+\eta \right)}^{2}}\right)E{\left\|z^{k}-z^{*}\right\|}_{2}^{2}. \end{array}$$

As $\beta_{k}>0,\lambda_{min}>0$, it holds that

$$\begin{array}{c} 1-\frac{\left(1-2\eta \right)\beta_{k}\lambda_{\min}\left(J_{C}^{T}J_{C}\right)}{2{\left(1+\eta \right)}^{2}}<1. \end{array}$$

Hence, the NSNGRK will gradually converge with the iteration. □

If the multi-fingered grasping problem has solutions, the NSNGRK method can definitely find the minimum-norm solution in the corresponding linear complementarity problem. Consequently, the NSNGRK method is applicable in all scenarios.

## 5. Numerical Experiments

We present the numerical solutions of our algorithm in five robot grasping scenarios: a 2D grasping problem, a 3D three-finger grasping problem with partial known finger forces, a 3D three-finger grasping problem with all unknown forces in two different configurations, and a 3D five-finger grasping problem.

All numerical experiments were conducted on a computer with a CPU of 2.30 GHz and 16.0 GB of memory. For the NSNGRK method, the zero vector was used as the initial value, with the termination criteria set to either exceeding 20,000 iterations or achieving a RES $<10^{-5}$, where RES is defined as the norm of the residual norm vector [23]

$$\mathrm{RES}\left(z^{\left(k\right)}\right)={\left\|\Phi \left(z^{\left(k\right)}\right)\right\|}_{2}.$$

To conduct a comparison with the NSNGRK method’s numerical solutions, we also provide results from the Pivot method in cases where they are applicable.

**Figure 3:**
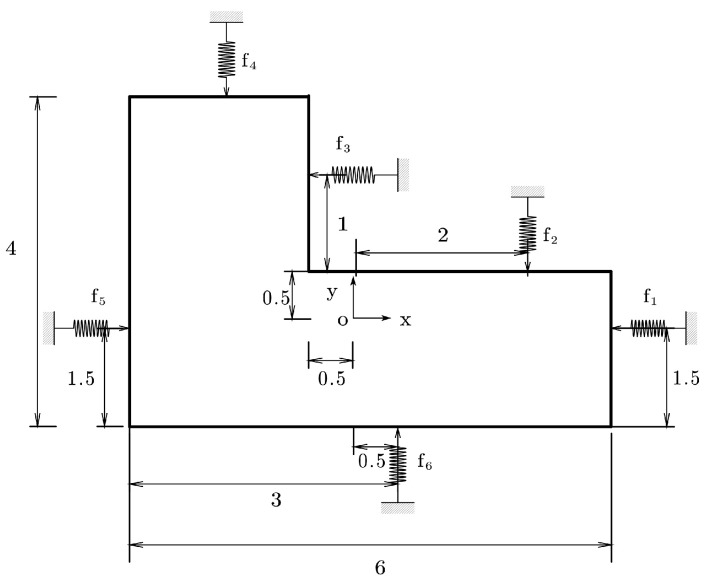
Configuration of Example 1.

**Example** **1.** *Consider a 2D grasping problem: a rigid planer object grasped by six frictionless elastic fingers [12]. The global coordinate system and finger contact locations are shown in Figure 3. The flexibility of the fingers is $E_{i}=0.1,i=1,\ldots ,6$. The external wrench is $P_{x}=-20,P_{y}=-30,M=-6$.*

The results of the Pivot method and the NSNGRK method are presented in Table 1 and Table 2. From both tables, it can be observed that the numerical solutions show complete agreement with the exact solutions within the error range. The results demonstrate that the NSNGRK method exhibits excellent performance in solving 2D grasping problems.

**Figure 4:**
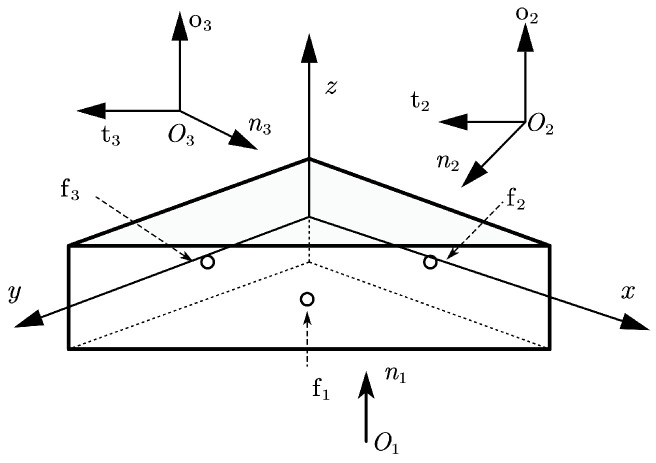
Configuration of Example 2.

**Example** **2.** *Consider a 3D grasping problem: an isosceles right-angle prism grasped by three fingers [38]. The global coordinate system and finger contact locations are shown in Figure 4. The positions of the contact points are $r_{1}={\left(-0.707,-0.707,0\right)}^{T}$, $r_{2}={\left(1,0,0\right)}^{T}$, $r_{3}={\left(0,1,0\right)}^{T}$. The friction coefficient is μ=0.3. The flexibility of the springs are, for the normal $E_{Ni}=1.5\times 10^{-6},i=1,2,3$, the tangential $E_{T1}=E_{T2}=6.0\times 10^{-6}$. Finger $f_{1}$ exerts a normal force $r_{N1}=1$ on the object.*

When approximating the friction cone with a pyramid, engineering standards require the pyramid to have at least four faces [39]. The normal and the tangential forces of fingers $f_{2}$ and $f_{3}$, the slippage displacements, and the object displacement calculated by the Pivot method and the NSNGRK method when approximating the friction cone with a four-face pyramid are given in Table 3 and Table 4, respectively. From these two tables, we can see that the results of the two methods are of the same order of magnitude. To improve friction cone representation, polyhedral cones with increased face counts were implemented. However, the Pivot method fails to converge when the face counts exceed eight, whereas the NSNGRK method successfully handles these cases. Table 5 shows the results of polyhedral approximations with 16 faces. Compared with Table 4, we find that normal force and tangential force of two fingers become closer, which shows that the NSNGRK method can obtain a more accurate solution with more faces and the NSNGRK method has more advantages than the Pivot method in solving larger-scale problems. On the other hand, we find that the grasping forces are enlarged with fewer pyramid faces.

**Figure 5:**
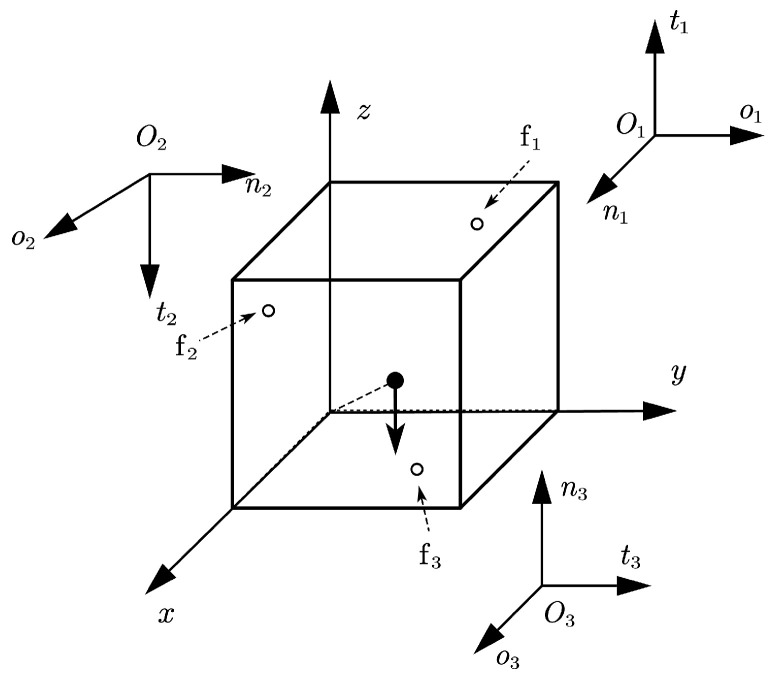
Configuration of Example 3.

**Example** **3.** *Consider a 3D grasping problem: a 1×1×1 cube grasped by three hard fingers [16]. The global coordinate system and finger contact locations are shown in Figure 5. The positions of the contact points are $r_{1}={\left(0.0,0.75,0.75\right)}^{T}$, $r_{2}={\left(0.75,0.0,0.75\right)}^{T}$, $r_{3}={\left(0.75,0.75,0.0\right)}^{T}$. The friction coefficient is 0.6. The gravity of the cube is 5.*

For the polyhedral approximations of a Coulomb friction cone with eight faces, the results calculated by two methods are shown in Table 6 and Table 7. When the number of faces is larger than eight, the Pivot method cannot solve the problem. The results of the NSNGRK method with 16 polyhedral faces are shown in Table 8.

In the existing three examples, the Pivot method can still yield results when there are fewer pyramid surfaces. However, in the following example, even with fewer pyramid surfaces, the Pivot method fails to produce results.

**Figure 6:**
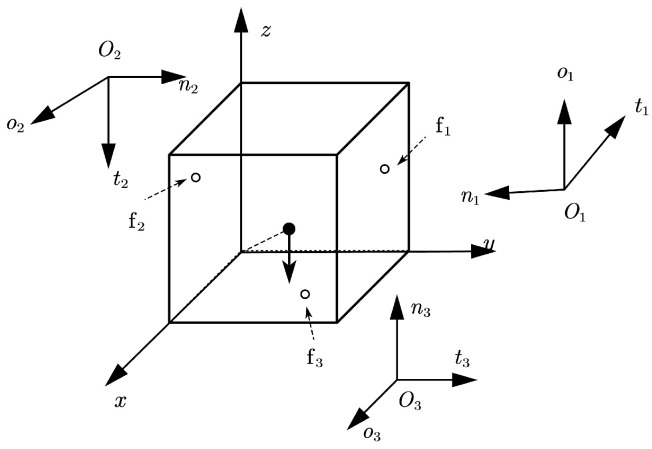
Configuration of Example 4.

**Example** **4.** *Consider another grasping configuration of a 1×1×1 cube grasped by three hard fingers, as shown in Figure 6. The finger and object contact locations are set to $p_{1}={\left(0.75,1.0,0.75\right)}^{T}$, $p_{2}={\left(0.75,0.0,0.75\right)}^{T}$, $p_{3}={\left(0.75,0.75,0.0\right)}^{T}$. The other parameters are set to the same values as in Example 3.*

In this example, the Pivot method fails to solve the problem. The results of the NSNGRK method are shown in Table 9 when the polyhedral approximation is set to six faces.

**Figure 7:**
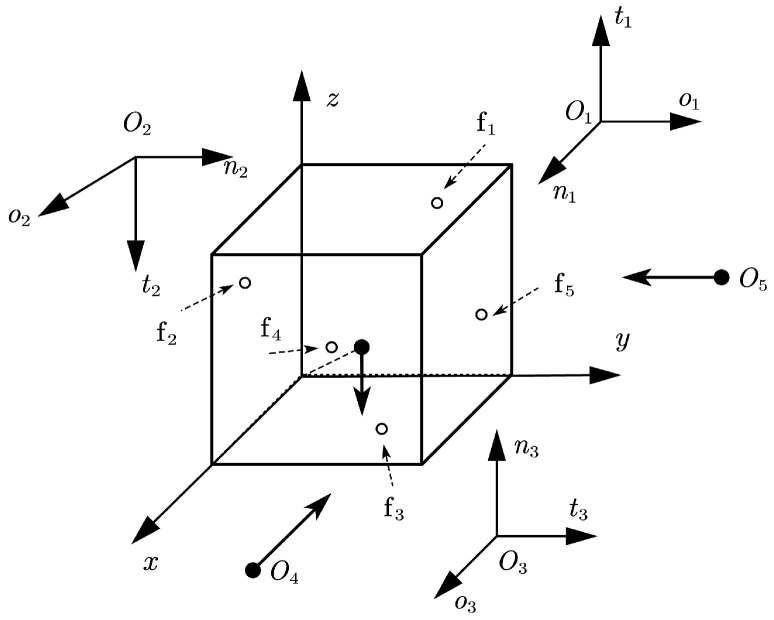
Configuration of Example 5.

**Example** **5.** *Consider a 1×1×1 cube grasped by five fingers, as shown in Figure 7. Finger $f_{4}$ and finger $f_{5}$ are frictionless, and the three others are hard fingers. The finger and object contact locations are set to $p_{1}={\left(0.0,0.75,0.75\right)}^{T}$, $p_{2}={\left(0.75,0.0,0.75\right)}^{T}$, $p_{3}={\left(0.75,0.75,0.0\right)}^{T}$, $p_{4}={\left(1.0,0.25,0.25\right)}^{T}$, $p_{5}={\left(0.25,1.0,0.25\right)}^{T}$. The other parameters are set to the same values as in Example 3.*

The Pivot method also fails to solve the problem in this example. The results of the NSNGRK method are shown in Table 10 when the polyhedral approximation is set to six faces. The five numerical experiments rigorously validate the proposed method’s computational feasibility and effectiveness across different multi-fingered grasping configurations. Although physical experiments are currently lacking, these numerical experiments are based on existing multi-fingered grasping problems, ensuring the numerical results can be realistically interpreted.

## 6. Conclusions

The multi-fingered grasping problem can be modeled as an LCP with a saddle-point coefficient matrix. To solve the problem, a Newton–subgradient non-smooth greedy randomized Kaczmarz method is proposed. The LCP is transformed into a non-smooth system of equations by using a merit function. Using subgradients to construct a generalized Jacobian matrix, we develop the subgradient non-smooth greedy randomized Kaczmarz method. The convergence of the proposed method is proven. The numerical results show its feasibility and effectiveness in solving LCPs arising from multi-fingered grasping problems.

## Figures and Tables

**Table 1:** Grasping forces and displacements for Example 1 when using the Pivot method.

Finger	Grasping Force	Fingertip Displacement	Object Displacement
1	$0.00000\times 10^{0}$	$-8.88178\times 10^{-16}$	$-2.00000\times 10^{0}$
2	$6.00000\times 10^{0}$	$6.00000\times 10^{-1}$	$-5.00000\times 10^{0}$
3	$0.00000\times 10^{0}$	$1.77636\times 10^{-15}$	$2.80000\times 10^{0}$
4	$0.00000\times 10^{0}$	$-3.55271\times 10^{-15}$	
5	$2.00000\times 10^{1}$	$2.00000\times 10^{0}$	
6	$3.60000\times 10^{1}$	$3.60000\times 10^{0}$	

**Table 2:** Grasping forces and displacements for Example 1 when using the NSNGRK method.

Finger	Grasping Force	Fingertip Displacement	Object Displacement
1	$-2.45271\times 10^{-6}$	$-2.45271\times 10^{-7}$	$-2.00000\times 10^{0}$
2	$5.99999\times 10^{0}$	$5.99999\times 10^{-1}$	$-5.00000\times 10^{0}$
3	$-3.60572\times 10^{-6}$	$-3.60572\times 10^{-7}$	$2.80000\times 10^{0}$
4	$-1.47403\times 10^{-6}$	$-1.47403\times 10^{-7}$	
5	$2.00000\times 10^{1}$	$2.00000\times 10^{0}$	
6	$3.60000\times 10^{1}$	$3.60000\times 10^{0}$	

**Table 3:** Grasping forces and displacements with 4 faces for Example 2 when using the Pivot method.

Finger	Grasping Force	Slippage Displacement	Object Displacement
2	$5.83270\times 10^{0}$	-	$7.42381\times 10^{-6}$
	$0.00000\times 10^{0}$	$0.00000\times 10^{0}$	$7.42381\times 10^{-6}$
	$-1.23730\times 10^{0}$	$0.00000\times 10^{0}$	$0.00000\times 10^{0}$
3	$5.83270\times 10^{0}$	-	$0.00000\times 10^{0}$
	$0.00000\times 10^{0}$	$0.00000\times 10^{0}$	$0.00000\times 10^{0}$
	$1.23730\times 10^{0}$	$0.00000\times 10^{0}$	$2.03240\times 10^{-21}$

**Table 4:** Grasping forces and displacements with 4 faces for Example 2 when using the NSNGRK method.

Finger	Grasping Force	Slippage Displacement	Object Displacement
2	$5.88661\times 10^{0}$	-	$8.37573\times 10^{-6}$
	$0.00000\times 10^{0}$	$0.00000\times 10^{0}$	$8.69457\times 10^{-6}$
	$-1.50224\times 10^{0}$	$0.00000\times 10^{0}$	$0.00000\times 10^{0}$
3	$5.40834\times 10^{0}$	-	$0.00000\times 10^{0}$
	$0.00000\times 10^{0}$	$0.00000\times 10^{0}$	$0.00000\times 10^{0}$
	$1.34281\times 10^{0}$	$0.00000\times 10^{0}$	$3.18846\times 10^{-7}$

**Table 5:** Grasping forces and displacements of NSNGRK with 16 faces for Example 2 when using the NSNGRK method.

Finger	Grasping Force	Slippage Displacement	Object Displacement
2	$5.46840\times 10^{0}$	-	$9.63920\times 10^{-6}$
	$0.00000\times 10^{0}$	$0.00000\times 10^{0}$	$9.64660\times 10^{-6}$
	$-1.60900\times 10^{0}$	$0.00000\times 10^{0}$	$0.00000\times 10^{0}$
3	$5.45730\times 10^{0}$	-	$0.00000\times 10^{0}$
	$0.00000\times 10^{0}$	$0.00000\times 10^{0}$	$0.00000\times 10^{0}$
	$1.60530\times 10^{0}$	$0.00000\times 10^{0}$	$7.40130\times 10^{-9}$

**Table 6:** Grasping forces and displacements with eight faces for Example 3 when using the Pivot method.

Finger	Grasping Force	Slippage Displacement	Object Displacement
1	$9.34263\times 10^{-1}$	-	$1.72378\times 10^{-6}$
	$2.14516\times 10^{-1}$	$-4.84598\times 10^{-6}$	$1.72256\times 10^{-6}$
	$5.17888\times 10^{-1}$	$-1.38596\times 10^{-5}$	$-2.15267\times 10^{-5}$
2	$9.34263\times 10^{-1}$	-	$9.18709\times 10^{-6}$
	$2.14516\times 10^{-1}$	$-4.84686\times 10^{-6}$	$-9.18709\times 10^{-6}$
	$-5.1788\times 10^{-1}$	$1.38575\times 10^{-5}$	$8.11268\times 10^{-10}$
3	$3.96422\times 10^{0}$	-	
	$-1.14878\times 10^{0}$	$0.00000\times 10^{0}$	
	$-1.14878\times 10^{0}$	$0.00000\times 10^{0}$	

**Table 7:** Grasping forces and displacements with 8 faces for Example 3 when using the NSNGRK method.

Finger	Grasping Force	Slippage Displacement	Object Displacement
1	$9.45155\times 10^{-1}$	-	$4.31116\times 10^{-6}$
	$1.97585\times 10^{-1}$	$-3.92507\times 10^{-6}$	$8.82936\times 10^{-7}$
	$5.23926\times 10^{-1}$	$-2.15251\times 10^{-5}$	$-2.56496\times 10^{-5}$
2	$9.45156\times 10^{-1}$	-	$4.45135\times 10^{-6}$
	$1.97585\times 10^{-1}$	$-2.72695\times 10^{-6}$	$-9.77932\times 10^{-6}$
	$-5.23926\times 10^{-1}$	$1.75292\times 10^{-5}$	$3.46273\times 10^{-6}$
3	$3.95215\times 10^{0}$	-	
	$-1.14274\times 10^{0}$	$0.00000\times 10^{0}$	
	$-1.14274\times 10^{0}$	$0.00000\times 10^{0}$	

**Table 8:** Grasping forces and displacements with 16 faces for Example 3 when using the NSNGRK method.

Finger	Grasping Force	Slippage Displacement	Object Displacement
1	$9.77217\times 10^{-1}$	-	$2.77735\times 10^{-6}$
	$1.143987\times 10^{-1}$	$-2.40058\times 10^{-6}$	$6.05216\times 10^{-6}$
	$5.75062\times 10^{-1}$	$-2.19914\times 10^{-5}$	$-3.14184\times 10^{-5}$
2	$9.77217\times 10^{-1}$	-	$1.14991\times 10^{-5}$
	$1.14387\times 10^{-1}$	$-1.16873\times 10^{-5}$	$-1.21088\times 10^{-5}$
	$-5.75062\times 10^{-1}$	$2.15342\times 10^{-5}$	$5.88634\times 10^{-6}$
3	$3.84988\times 10^{0}$	-	
	$-1.09160\times 10^{0}$	$0.00000\times 10^{0}$	
	$-1.09160\times 10^{0}$	$0.00000\times 10^{0}$	

**Table 9:** Grasping forces and displacements of NSNGRK with 6 faces for Example 4.

Finger	Grasping Force	Slippage Displacement	Object Displacement
1	$2.43084\times 10^{0}$	-	$1.95578\times 10^{-5}$
	$4.76666\times 10^{-6}$	$-4.76666\times 10^{-6}$	$2.25914\times 10^{-5}$
	$1.94984\times 10^{-5}$	$-1.94984\times 10^{-5}$	$-8.64359\times 10^{-6}$
2	$2.78653\times 10^{0}$	-	$1.11060\times 10^{-5}$
	$1.11621\times 10^{-5}$	$-1.11621\times 10^{-5}$	$-1.03610\times 10^{-5}$
	$-1.43136\times 10^{0}$	$1.42673\times 10^{-5}$	$2.94105\times 10^{-5}$
3	$3.20750\times 10^{0}$	-	
	$-1.66667\times 10^{0}$	$0.00000\times 10^{0}$	
	$-3.55692\times 10^{-1}$	$0.00000\times 10^{0}$	

**Table 10:** Grasping forces and displacements of the NSNGRK method with 6 faces for Example 5.

Finger	Grasping Force	Slippage Displacement	Object Displacement
1	$1.14604\times 10^{0}$	-	$2.53391\times 10^{-7}$
	$1.21074\times 10^{0}$	$-8.82529\times 10^{-6}$	$2.12692\times 10^{-6}$
	$6.74767\times 10^{-1}$	$-1.27932\times 10^{-5}$	$-2.49392\times 10^{-5}$
2	$1.07365\times 10^{0}$	-	$1.48451\times 10^{-5}$
	$1.00729\times 10^{-1}$	$-1.57578\times 10^{-6}$	$-2.23744\times 10^{-6}$
	$-6.36257\times 10^{-1}$	$2.23067\times 10^{-5}$	$1.43231\times 10^{-6}$
3	$3.68898\times 10^{0}$	-	
	$-8.85082\times 10^{-1}$	$0.00000\times 10^{0}$	
	$-7.01790\times 10^{-1}$	$0.00000\times 10^{0}$	
4	$1.60226\times 10^{-1}$	-	
5	$4.92930\times 10^{-1}$	-	

## Data Availability

The data are contained within the article.
